# Domestic violence: Screening and management in South Africa

**DOI:** 10.4102/safp.v67i1.6000

**Published:** 2025-01-06

**Authors:** Deidré Pretorius, Aviva Ruch

**Affiliations:** 1 Division of Family Medicine, School of Clinical Medicine, Faculty of Health, University of the Witwatersrand, Johannesburg, South Africa; 2 Unit for Undergraduate Medical Education, Faculty of Health, University of the Witwatersrand, Johannesburg, South Africa

**Keywords:** domestic violence, abuse, legislation, gender-based violence, intimate partner violence

## Abstract

Violence manifests in various ways in healthcare, including trauma from an undifferentiated patient, psychosomatic illness, substance abuse or dependency and mental health challenges. Different forms of violence exist, such as intimate partner violence, gender-based violence, domestic violence, child abuse, neglect, elder abuse, sexual violence, self-directed violence and collective violence. These may be included in domestic violence or exist as standalone forms. Health practitioners play a pivotal role in managing incidents of domestic violence. This article highlights the definitions in the *Amended Domestic Violence Act* of 2021 and suggests screening options for domestic violence. The authors also suggest screening tools, a management flow diagram and contact numbers for resources. Domestic violence can be a generational curse that compromises biopsychosocial wellbeing. To break the perceived culture of violence, healthcare workers play a pivotal role in screening and management, as well as the mandatory reporting of domestic violence when children and the elderly are sharing such a household.

## Introduction

Violence within homes between people who care for each other is confusing and terrifying for both those involved and their health professionals. Violence manifests in numerous ways in healthcare such as trauma or an undifferentiated patient, psychosomatic illness, substance abuse or dependency and/or mental health challenges. Different forms of violence exist such as intimate partner violence, gender-based violence, domestic violence, child abuse, neglect, elder abuse, sexual violence, self-directed violence and collective violence that may be included in domestic violence or standalone forms of violence.^1^ Apart from the statutory obligations of health practitioners, they are also pivotal in managing incidents of domestic violence. Practitioners must create awareness, screen for and identify abuse, assess and document incidents and provide emotional support.^2^ In addition, they must develop safety plans, offer medical treatment and referrals, ensure continuous support and follow-up and encourage victim-survivors to report abuse.^2^

It is often difficult to figure out the cause or consequence of this complex interplay between power and vulnerability. A systematic review indicated that women who experienced violence as a child (either violence in the family of origin or adult victim-survivor of child abuse) are often more vulnerable, economically dependent and have a lack of social support and thus, are more likely to experience violence in the household.^3,4^ Emotional dependency in men is a risk for violence.^3,4^ Women with disabilities are more likely to be abused than women without disabilities.^5^ Numerous research articles link alcohol use to emotional dysregulation and impaired judgement, which often leads to domestic violence.^6^ There may also be a greater risk for women using marijuana to perpetrate violence if they have a history of perpetrating violence before marriage.^7^ Mshweshwe^8^ postulated that domestic violence is a ‘consequence of the complex interplay of patriarchy, culture, and the negative masculine construct’. Not only does violence cause trauma and even death, it also affects the quality of life of families, and has a wide range of physical and mental sequelae.^9^

Women exposed to domestic violence are more likely to present with sleep challenges, headaches, gastrointestinal disorders or depression.^10^ A study in the United States identified intimate partner problems in 26% (*n* = 1327) of suicide cases and 43% of these individuals experienced relational problems before attempting suicide.^11^ For both men and women, mental health issues such as depression and anxiety are statistically associated with domestic violence.^1,9^ Physical and mental conditions are not the only sequelae of domestic violence. Sexual and reproductive health, low birthweight babies and increased risk for human immunodeficiency virus (HIV) are also noted consequences of domestic violence resulting in poorer health outcomes for women with chronic conditions.^12,13^ Primary care services can play a crucial role in identifying and managing patients experiencing domestic violence appropriately. The current statutory obligation under the new *Domestic Violence Amendment Act* trumps recommendations in national guidelines and policies as they predate the amendment act. Despite this, the South African National guideline concerning the empowerment of victim-survivors of violence encourages all services to recognise and respond to vulnerable individuals affected by violence and abuse.^14^ The Health Professions Council of South Africa is clear that domestic violence is not a private matter and health professionals must screen, assess, manage and refer appropriately.^15^ The South African Maternal Perinatal and Neonatal Health Policy (2021),^16^ and various legislations mandate that healthcare professionals manage domestic violence.^17,18,19^

Health professionals often are focussed on the visible presentation of domestic violence and have limited knowledge and understanding about the scope and complexity of what legislation defines as domestic violence. For the purpose of this continuous medical education article, we want to suggest an approach to identify and manage domestic violence as defined by law in South Africa.^19,20^

## Definitions as described in law

**FIGURE 1:**
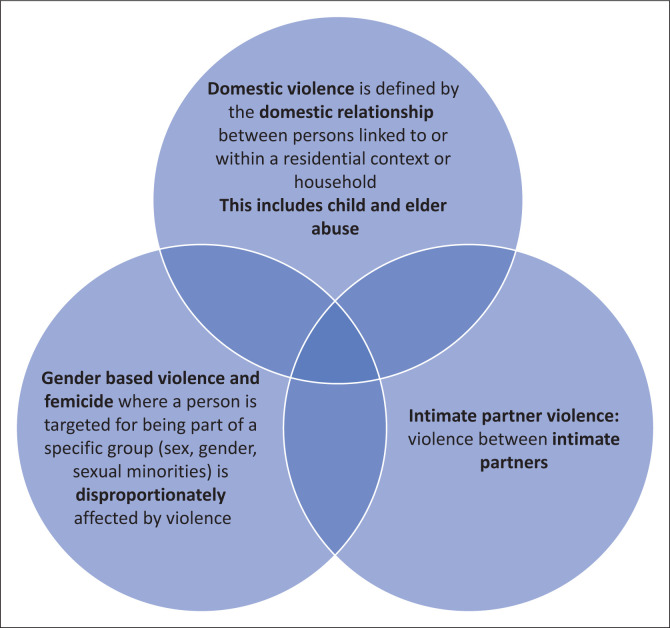
The overlap between leading forms of violence occurring in a household.

There is often confusion between the terms domestic, gender-based and intimate partner violence (IPV).^21^ Figure 1 illustrates how these three concepts may overlap within a household. Domestic violence is violent or aggressive behaviour within the home, typically involving the violent abuse of a spouse or partner but can involve other members of the household. In the past, child abuse and elder abuse were covered under its own legislation. The amended *Domestic Violence Act of 2021* explicitly includes child and elderly abuse and thus, complements existing legislation for these two groups.^19^ Therefore, domestic violence in this article includes violence against children, the elderly and vulnerable members of a household.^19,20^

To screen for and manage domestic violence, it is important to know the relevant basic legal definitions. A complainant is:

[*A*]ny person who is or has been in a domestic relationship with a respondent and who is or has been subjected or allegedly subjected to an act of domestic violence, including any child in the care of the complainant.^19,20^

A respondent is ‘any person who is or has been in a domestic relationship with a complainant and who has committed or allegedly committed an act of domestic violence against the complainant’ (Box 1).^19,20^

BOX 1 Domestic relationship according to South African legislation.

A domestic relationship means ‘a relationship between a complainant and a respondent’ in any of the following ways:

- they are or were married to each other, including marriage according to any law, custom or religion
- they (whether they are of the same or of the opposite sex) live or lived together in a relationship in the nature of marriage, although they are not, or were not, married to each other, or are not able to be married to each other
- they are the parents of a child or are persons who have or had parental responsibility for that child (whether or not at the same time)
- they are family members related by consanguinity, affinity or adoption
- they are or were in an engagement, dating or customary relationship, including an actual or perceived romantic, intimate or sexual relationship of any duration
- they are persons in a close relationship that share or [recently] shared the same residence.

*Source*: Domestic Violence Amendment, Act No 4 of 2021: Gazette 45824 of 28 January 2022. 2022 [cited 2024 Jan 8]. Available from: https://www.parliament.gov.za/storage/app/media/Acts/2021/Act_No_14_of_2021_Domestic_Violence_Amendment_Act.pdf; Domestic Violence Act 116 of 1998 | South African Government. 1998 [cited 2024 Jan 8]. Available from: https://www.gov.za/sites/default/files/gcis_document/201409/a116-980.pdf

The law defines a ‘close relationship’ as a degree of trust that exists between two persons who have a level of dependence on, and commitment to, the other person.^19,20^ They also have frequent contact and there is a degree of intimacy between them. It is no secret that South Africa has some of the highest levels of gender-based violence in the world and therefore the socio-political emphasis is on protection and empowerment of women. However, men and persons from sexually diverse or minority groups are also victims of domestic violence.^22,23,24,25^ The prevalence of IPV between same-sex couples is just as high as in heterosexual couples.^26^ The Centres of Disease Control in the United States reported that 47.3% (*n* = 59 million) women and 44.2% (*n* = 52.1 million) men were exposed to domestic and sexual violence over their lifetime.^27^ In a South African study of 3048 men and women in sexual minority groups, nearly 70.5% reported to police witnessing physical violence against people in same-sex relationships.^25^ This does not include verbal abuse, bullying and sexual violence or abuse not reported to law enforcement.^25^ According to the second quarter of 2023 crime statistics in South Africa, the police recorded 10 516 rapes, 1514 cases of attempted murder, 881 women murdered and 14 401 assaults against female victim-survivors in July, August and September.^28^

## Screening and management

Domestic violence is a criminal offence and there can be no reason for the justification of abuse. It is important to show empathy and demonstrate a non-judgemental attitude to all victim-survivors of abuse.

Several tools exist to assist practitioners in identifying and documenting domestic violence namely, HITS (Hurt, Insult, Threaten and Screen), Woman Abuse Screening Tool (WAST), Partner Violence Screen (PVS) and Abuse Assessment Scale (AAS).^29,30,31,32^ Saimen et al.^33^ found that the WAST two-question tool^34^ developed for family practice had a sensitivity of 45.2% and specificity of 98% in the South African context. Overall, these tools use various psychometric parameters to assess risk, but there is no one tool that has been found to be superior to the others.^30^ Furthermore, the focus of these questionnaires, with the exception of the AAS tool, is mainly on physical and verbal abuse. Considering the several types of domestic violence specified in the South African legislation (see Box 2), it can be missed if only these tools are used.

BOX 2 Types of domestic violence according to South African legislation.

**Types of domestic violence:**

- Coercive behaviour means to compel or force a complainant to abstain from doing anything that they have a lawful right to do or to force them to do anything that they have a lawful right to abstain from doing.
- Controlling behaviour is depriving and/or isolating an individual from sources of support, exploiting their resources or capacities for personal gain, depriving them of the means needed for independence, resistance or escape, or regulating their everyday behaviour.
- Economic abuse – if the complainant suffers financial damages caused by the respondent, for example, where the respondent sells household property or uses a joint bank account for personal use without the consent of the complainant.
- Elder abuse means abuse of an older person as contemplated in section 30(2) of the *Older Persons Act, 2006* (Act No. 13 of 2006)^7^, occurring within a domestic relationship [Mandatory reporting].
- Emotional and psychological abuse – if the respondent verbally insults or humiliates the complainant; it includes degrading, manipulating, threatening, offensive, intimidating or humiliating conduct towards a complainant that causes mental or psychological harm to a complainant, including insults, ridicule or name calling; threats to cause emotional pain; exhibition of obsessive possessiveness or jealousy, which constitutes a serious invasion of the complainant’s privacy, liberty, integrity or security; the wilful damaging or destruction of any property in close vicinity of the person, harm or threaten to harm a household pet or other animal, whose welfare affects a complainant’s wellbeing; to disclose or threaten to disclose a complainant’s sexual orientation; threaten the complainant with the death or injury of another person or damage of another person’s property; or threats to commit suicide or self-harm.
- Exposing a child to domestic violence – intentionally cause a child to see or hear domestic violence; or experience the effects of domestic violence (*Children’s Act 38 of 2005*^8^ and its subsequent amendments also apply) [Mandatory reporting].
- Intimidation, harassment – the unreasonable following, watching, stalking, pursuing or accosting of the complainant or a related person;
  loitering outside of or near the building or place where the complainant or a related person resides, works, carries on business, studies or happens to be; to repeatedly contact the complainant by means of an electronic communications service, or has unauthorised access to a complainant’s communication or electronic communication; monitoring or tracking of the complainant’s movements, activities or interpersonal associations without consent, including, for example, by using technology; enter any part of the joint residence that is exclusively used by the complainant or other property of the complainant, without permission; to disclose an electronic communication to the complainant, or cause the complainant to receive a communication, which is abusive, degrading, offensive or humiliating; violates or offends the sexual integrity or dignity of a complainant; or inspires the belief in the complainant that they or a related person may be harmed, or their property may be damaged.
- Physical abuse – if the complainant is being physically injured by the respondent, for example, being punched, kicked, burned or pushed.
- Property damages – if the respondent damages any property that belongs to the complainant.
- Related person abuse – threats to cause physical violence to, or damage the property of, a related person (any member of the family or household or a person in a close relationship).
- Sexual abuse – means any conduct that abuses, humiliates, degrades or otherwise violates the sexual integrity of the complainant, irrespective of whether such conduct constitutes a sexual offence as contemplated in the Criminal Law (*Sexual Offences and Related Matters) Amendment Act, 2007* (Act No. 32 of 2007).
- Sexual harassment – unwelcome sexual attention, unwelcome explicit or implicit behaviour, suggestions, gestures, remarks made, communications sent or delivered; electronic or communication of sexual nature or regarding the complainant’s or related person’s sexual orientation, gender or gender expression that is offending, intimidating or humiliating. It also includes an implied or expressed promise of reward if they comply with a sexually oriented request.
- Spiritual abuse means advocating hatred against the complainant because of their religious or spiritual beliefs, which constitutes incitement to cause harm to the complainant; preventing the complainant from exercising their constitutional right to freedom of conscience, religion, thought, belief and opinion, including to give external manifestation to their religious or spiritual convictions and beliefs; or manipulating the complainant’s religious or spiritual convictions and beliefs to justify or rationalise abusing the complainant.

*Source*: Domestic Violence Amendment, Act No 4 of 2021: Gazette 45824 of 28 January 2022. 2022 [cited 2024 Jan 8]. Available from: Domestic Violence Act 116 of 1998 | South African Government. 1998 [cited 2024 Jan 8]. Available from: https://www.gov.za/sites/default/files/gcis_document/201409/a116-980.pdf

### Possible screening questions for domestic violence

When a patient presents with injuries, say, ‘When I see injuries like this, I know it is not accidental – who is hurting you?’ The best way to screen in the absence of physical evidence or disclosure is to ask ‘How are relationships at home’; ‘Any challenges at home?’; ‘How do you and other family members cope with these challenges?’ and ‘Stressors can lead to conflict … how do you resolve conflict?’ Non-verbal communication must also be scrutinised and reflected on, especially if there is any deviation from the usual pattern. The onset of physical complaints with no diagnostic pattern can also alert a doctor to screen for domestic violence. If not sure, the doctor can use generalisations such as ‘We know domestic violence affects a lot of people in South Africa, therefore I ask all the patients if they have witnessed or experienced any form of violence?’ The doctor can reflect: ‘You say your husband gets very irritated when you say you are tired … what does he say or do when he’s irritated?’ or ‘… when you are tired, does he call you names or physically or sexually hurt you?’ Using the WAST tool ask (1) ‘How does the patient in general describe their relationship with a partner’ (lots of tension, some tension or no tension); (2) ‘Do you and your partner work out arguments with …?’ (Great difficulty, some difficulty or no difficulty). It is noteworthy that these two questions assume that all abuse is triggered by arguments with partners and do not represent the broader concepts described in our context and legislation.

**FIGURE 2:**
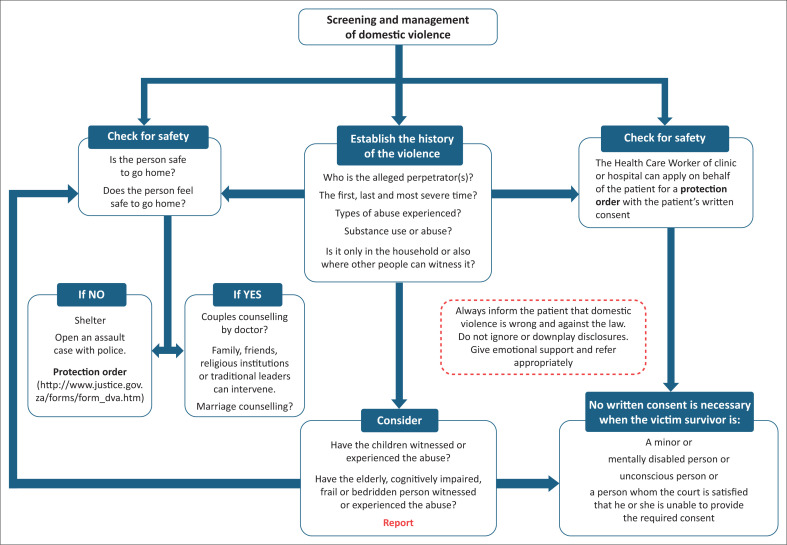
Flow diagram for screening and management of domestic violence.

Figure 2 can aid you in managing patients once they screen positive for being at risk for domestic violence and Box 3 has useful resources.

BOX 3 Resources.

**Available resources:**

- Simplified guide to domestic violence and the protection order
https://www.saferspaces.org.za/uploads/files/Domestic_Violence_Guide.pdf
- **Protection order** is free. Forms to complete at police station, magistrate’s office or on-line: http://www.justice.gov.za/forms/form_dva.htm (application can also be made on-line)
- **POWA** provides counselling, both over the phone and in person, temporary shelter for and legal help to women who have experienced violence.
  Website: http://www.powa.co.za Tel: 011 642 4345/6E-mail: info@powa.co.za (link sends e-mail). Counselling service via email: counselling@powa.co.za
- **TEARS Foundation** provides access to crisis intervention, advocacy, counselling and prevention education services for those impacted by domestic violence, sexual assault and child sexual abuse. Website: http://www.tears.co.za/ (link is external). Free SMS helpline: *134*7355#
  Tel: 010 590 592024/7 Emergency Helpline: 0800 083 277
  Email: info@tears.co.za (link sends e-mail)

**Helplines**

- SAPS Emergency 10111
- Gender-Based Violence Command Centre 0800 428 428
- STOP Gender Violence Helpline 0800 150 150/ *120*7867#
- Halt Elder Abuse Line (Heal) Helpline: 0800003081 E-mail: action@actiononelderabusesa.co.za (link sends e-mail)

POWA, people opposing women abuse; SAPS, South African Police Services.

When a person wants to apply for a protection order, it is important to note that photos of the abuse or injuries, receipts or photos of items that were damaged or sold, statements of people who witnessed the abuse, confirming letters from social workers, psychologists or other healthcare workers (including the official J88 for documentation of injuries for legal purposes) can make the case stronger, but it is not a requirement in obtaining a protection order.^35^

## Conclusion and take-home message

Domestic violence can be a generational curse compromising biopsychosocial wellbeing. To break the perceived culture of violence, healthcare workers play a pivotal role in screening, management and the mandatory reporting of domestic violence, especially when children or the elderly are involved in such households.

## References

[1] Krug EG, Mercy JA, Dahlberg LL, Zwi AB. The world report on violence and health. Lancet Lond Engl. 2002;360(9339):1083–1088. 10.1016/S0140-6736(02)11133-0 12384003

[2] Moreira DN, Pinto da Costa M. The role of family doctors in the management of domestic violence cases – A qualitative study in Portugal. BMC Health Serv Res. 2023;23(1):571. 10.1186/s12913-023-09501-9 37268919 PMC10237072

[3] Pereira ME, Azeredo A, Moreira D, Brandão I, Almeida F. Personality characteristics of victims of intimate partner violence: A systematic review. Aggress Violent Behav. 2020;52:101423. 10.1016/j.avb.2020.101423

[4] Bornstein RF. The complex relationship between dependency and domestic violence: Converging psychological factors and social forces. Am Psychol. 2006;61(6):595–606. 10.1037/0003-066X.61.6.595 16953747

[5] Stern E, Van der Heijden I, Dunkle K. How people with disabilities experience programs to prevent intimate partner violence across four countries. Eval Program Plann. 2020;79:101770. 10.1016/j.evalprogplan.2019.101770 31865010

[6] Grigorian HL, Brem MJ, Garner A, Florimbio AR, Wolford-Clevenger C, Stuart GL. Alcohol use and problems as a potential mediator of the relationship between emotion dysregulation and intimate partner violence perpetration. Psychol Violence. 2020;10(1):91–99. 10.1037/vio0000237 33224553 PMC7678751

[7] Smith PH, Homish GG, Collins RL, Giovino GA, White HR, Leonard KE. Couples’ marijuana use is inversely related to their intimate partner violence over the first 9 years of marriage. Psychol Addict Behav J Soc Psychol Addict Behav. 2014;28(3):734–742. 10.1037/a0037302 PMC4282761 25134048

[8] Mshweshwe L. Understanding domestic violence: Masculinity, culture, traditions. Heliyon. 2020;6(10):E05334. 10.1016/j.heliyon.2020.e05334 33150213 PMC7599123

[9] Oram S, Fisher HL, Minnis H, et al. The Lancet Psychiatry commission on intimate partner violence and mental health: Advancing mental health services, research, and policy. Lancet Psychiatry. 2022;9(6):487–524. 10.1016/S2215-0366(22)00008-6 35569504

[10] Sugg N. Intimate partner violence: Prevalence, health consequences, and intervention. Med Clin North Am. 2015;99(3):629–649. 10.1016/j.mcna.2015.01.012 25841604

[11] Brown S, Seals J. Intimate partner problems and suicide: Are we missing the violence?. J Inj Violence Res. 2019;11(1):53–64. 10.5249/jivr.v11i1.997 30636256 PMC6420923

[12] Stubbs A, Szoeke C. The effect of intimate partner violence on the physical health and health-related behaviors of women: A systematic review of the literature. Trauma Violence Abuse. 2022;23(4):1157–1172. 10.1177/1524838020985541 33541243

[13] Enaifoghe A, Dlelana M, Durokifa AA, Dlamini NP. The prevalence of gender-based violence against women in South Africa: A call for action. Afr J Gend Soc Dev Former J Gend Inf Dev Afr. 2021;10(1):117–146. 10.31920/2634-3622/2021/v10n1a6

[14] National_policy_guidelines_for_victim_empowerment.pdf [homepage on the Internet]. 2009 [cited 2024 Jun 14]. Available from: https://www.ohchr.org/sites/default/files/Documents/Issues/Women/SR/Shelters/National_policy_guidelines_for_victim_empowerment.pdf

[15] Booklet_8_Reproductive_Health_September_2016.pdf [homepage on the Internet]. 2016 [cited 2024 Jun 14]. Available from: https://www.hpcsa.co.za/Uploads/professional_practice/ethics/Booklet_8_Reproductive_Health_September_2016.pdf

[16] The South African Maternal Perinatal and Neonatal Health Policy [homepage on the Internet]. 2021 [cited 2024 Jun 14]. Available from: https://knowledgehub.health.gov.za/elibrary/south-african-maternal-perinatal-and-neonatal-health-policy

[17] Older Persons Act, 2006, No. 13 of 2006: 1 April 2010: Gazette 33075 of 1 April 2010. 2010 [cited 2024 Jun 14]. Available from: https://www.gov.za/sites/default/files/gcis_document/201409/a41-070.pdf

[18] Children’s Amendment Act, 2007, No. 41 of 2007: Gazette 33076 of 1 April 2010. 2010 [cited 2024 Jun 14]. Available from: https://www.gov.za/documents/childrens-amendment-act-commencement

[19] Domestic Violence Amendment, Act No 4 of 2021: Gazette 45824 of 28 January 2022. 2022 [cited 2024 Jan 8]. Available from: https://www.parliament.gov.za/storage/app/media/Acts/2021/Act_No_14_of_2021_Domestic_Violence_Amendment_Act.pdf

[20] Domestic Violence Act 116 of 1998 | South African Government. 1998 [cited 2024 Jan 8]. Available from: https://www.gov.za/sites/default/files/gcis_document/201409/a116-980.pdf

[21] Wilkins N, Tsao B, Davis R, Hertz M, Klevens J. Connecting the dots: An overview of the links among multiple forms of violence [homepage on the Internet]. Centers for Disease Control and Prevention and Prevention Institute; 2014 [cited 2024 Jan 9]. Available from: https://stacks.cdc.gov/view/cdc/31552

[22] Thobejane TD, Luthada V. An investigation into the trend of domestic violence on men: The case of South Africa [serial Online]. OIDA Int J Sustain Dev Ont Int Dev Agency. 2019 [cited 2024 Feb 12];12(03):12–18. Available from: https://oidaijsd.com/wp-content/uploads/2019/08/12-03-01.pdf

[23] Thusi X, Mlambo VH. South Africa’s gender-based violence: An exploration of a single sided account. EUREKA Soc Humanit. 2023;(2):73–80. 10.21303/2504-5571.2023.002734

[24] Closson K, Zulu B, Jesson J, et al. Examining gender and sexual orientation differences in physical intimate partner violence experienced and perpetrated by youth living in eThekwini district South Africa during the COVID-19 pandemic. BMC Public Health. 2023;23(1):2300. 10.1186/s12889-023-17199-x 37990170 PMC10664660

[25] Abaver DT, Cishe EN. Violence, abuse and discrimination: Key factors militating against control of HIV/AIDS among the LGBTI sector. SAHARA J J Soc Asp HIVAIDS Res Alliance. 2018;15(1):60–70. 10.1080/17290376.2018.1492960 PMC6060376 30025496

[26] Rollè L, Giardina G, Caldarera AM, Gerino E, Brustia P. When intimate partner violence meets same sex couples: A review of same sex intimate partner violence. Front Psychol. 2018;9. 10.3389/fpsyg.2018.01506 PMC6113571 30186202

[27] Leemis W. The National Intimate Partner and Sexual Violence Survey: 2016/2017 Report on intimate partner violence [homepage on the Internet]. Centers for Disease Control and Prevention; 2022 [cited 2024 Feb 12]. Available from: https://www.cdc.gov/nisvs/documentation/nisvsreportonipv_2022.pdf?CDC_AAref_Val=https://www.cdc.gov/violenceprevention/pdf/nisvs/nisvsreportonipv_2022.pdf

[28] Mpako A, Ndoma S. South Africans see gender-based violence as most important women’s-rights issue to address [homepage on the Internet]. 2023 [cited 2024 Feb 12]. Available from: https://www.afrobarometer.org/wp-content/uploads/2023/11/AD738-South-Africans-see-gender-based-violence-as-a-top-priority-Afrobarometer-24nov23.pdf

[29] Woman Abuse Screening Tool (WAST) [homepage on the Internet]. MDCalc. [cited 2024 May 3]. Available from: https://www.mdcalc.com/calc/10396/woman-abuse-screening-tool-wast

[30] Rabin RF, Jennings JM, Campbell JC, Bair-Merritt MH. Intimate partner violence screening tools. Am J Prev Med. 2009;36(5):439–445.e4. 10.1016/j.amepre.2009.01.024 19362697 PMC2688958

[31] Abuse-Assessment-Screen-_AAS_.pdf [homepage on the Internet]. [cited 2024 May 3]. Available from: https://chipts.ucla.edu/wp-content/uploads/downloads/2012/01/Abuse-Assessment-Screen-_AAS_.pdf

[32] Rabin RF, Jennings JM, Campbell JC, Bair-Merritt MH. Intimate partner violence screening tools: A systematic review. Am J Prev Med [serial online]. 2009 [cited 2024 Feb 12];36(5):439–445.e4. 10.1016/j.amepre.2009.01.024 19362697 PMC2688958

[33] Saimen A, Armstrong E, Manitshana C, Govender I. Evaluation of a two-question screening tool in the detection of intimate partner violence in a primary healthcare setting in South Africa. South Afr Fam Pract. 2016;58(5):74. 10.4102/safp.v58i5.4588

[34] Brown JB, Lent B, Brett PJ, Sas G, Pederson LL. Development of the woman abuse screening tool for use in family practice [serial online]. Fam Med. 1996 [cited 2024 Feb 12];28(6):422–428. Available from: https://pubmed.ncbi.nlm.nih.gov/8791071/8791071

[35] Steps to obtain a Protection Order. Family and Divorce law in South Africa - A comprehensive guide. divorcelaws. 2024 [cited 2024 Aug 27]. Available from: https://www.divorcelaws.co.za/steps-to-obtain-a-protection-order.html

